# Estrogen Signaling Modulates Allergic Inflammation and Contributes to Sex Differences in Asthma

**DOI:** 10.3389/fimmu.2015.00568

**Published:** 2015-11-16

**Authors:** Aleksander Keselman, Nicola Heller

**Affiliations:** ^1^Department of Anesthesiology and Critical Care Medicine, Johns Hopkins University School of Medicine, Baltimore, MD, USA

**Keywords:** estrogen, allergy, asthma, inflammation, sex difference, macrophages, IL-4 and IL-13

## Abstract

Asthma is a chronic airway inflammatory disease that affects ~300 million people worldwide. It is characterized by airway constriction that leads to wheezing, coughing, and shortness of breath. The most common treatments are corticosteroids and β2-adrenergic receptor antagonists, which target inflammation and airway smooth muscle constriction, respectively. The incidence and severity of asthma is greater in women than in men, and women are more prone to develop corticosteroid-resistant or “hard-to-treat” asthma. Puberty, menstruation, pregnancy, menopause, and oral contraceptives are known to contribute to disease outcome in women, suggesting a role for estrogen and other hormones impacting allergic inflammation. Currently, the mechanisms underlying these sex differences are poorly understood, although the effect of sex hormones, such as estrogen, on allergic inflammation is gaining interest. Asthma presents as a heterogeneous disease. In typical Th2-type allergic asthma, interleukin (IL)-4 and IL-13 predominate, driving IgE production and recruitment of eosinophils into the lungs. Chronic Th2-inflammation in the lung results in structural changes and activation of multiple immune cell types, leading to a deterioration of lung function over time. Most immune cells express estrogen receptors (ERα, ERβ, or the membrane-bound G-protein-coupled ER) to varying degrees and can respond to the hormone. Together these receptors have demonstrated the capacity to regulate a spectrum of immune functions, including adhesion, migration, survival, wound healing, and antibody and cytokine production. This review will cover the current understanding of estrogen signaling in allergic inflammation and discuss how this signaling may contribute to sex differences in asthma and allergy.

## Introduction: Background on Allergic Inflammation and Asthma

Allergic disorders are characterized by Th2 responses against innocuous environmental factors that result in eosinophilic inflammation and the production of interleukin (IL)-4, IL-13, and IgE. Genetic, behavioral, sex-specific, and environmental factors contribute to the rising incidence of allergic diseases. Environmental factors that contribute to allergies are extremely broad and include a variety of food, plant, insect, and pollutant products. Allergic sensitization initiates the “atopic march,” and chronic exposure to environmental insults at epithelial surfaces leads to the development of a spectrum of allergic disorders ranging from dermatitis to asthma [reviewed in Ref. (1, 2)]. Allergic diseases are increasingly common in the Western world and to date have no cure. Current treatments broadly include antihistamines, anticongestants, antileukotrienes, bronchodilators, and steroids. Th2 (IL-4, IL-13 and IL-5) cytokine-neutralizing antibodies are currently in phase II and phase III of clinical trials with promising results (3–6). However, hard-to-treat allergies limit the effectiveness of current therapies, especially in the case of asthma.

Asthma is an allergic inflammation of the respiratory tract that affects >300 million people worldwide. It has a prevalence of 1 in 12 adults and 1 in 11 children (7, 8). Respiratory inflammation associated with asthma leads to fibrosis and airway constriction, which presents as wheezing and shortness of breath. The epithelium serves as the interface between allergens and the underlying immune system and thus initiates inflammation by secreting cytokines and chemokines such as IL-5, regulated on activation, normal T cell expressed and secreted (RANTES or CCL5), IL-22, IL-25, thymic stromal lymphopoietin (TSLP), and IL-33 [reviewed in Ref. (9)]. Th2-polarized T cells are abundant in the draining lymph nodes of the lungs, circulating blood, and bronchoalveolar lavage (BAL) of patients with asthma- and allergen-challenged mice. They are central to orchestrating inflammation by secreting cytokines, such as IL-4, IL-5, IL-13, and granulocyte macrophage colony-stimulating factor (GM-CSF). The BAL of asthmatic patients and allergen-challenged mice contains abundant eosinophils and M2-polarized macrophages, which together secrete a variety of inflammation-promoting factors (10–12) [reviewed in Ref. (13)]. These factors include chemokines that recruit more lymphocytes into the airways, prostaglandins that promote bronchoconstriction, and matrix metalloproteinases (MMPs) that promote fibrotic tissue remodeling. Histologically, the airways are infiltrated by leukocytes, and the surrounding smooth muscle is thickened from enhanced collagen deposition. In hard-to-treat cases of steroid-resistant asthma, neutrophils infiltrate the BAL as a result of Th17 responses and IL-8 production.

Asthma incidence and severity exhibit sex differences, and there is mounting clinical and animal model evidence that implicates sex hormones, particularly estrogen, in being key mediators of these differences [reviewed in Ref. (14)]. In the following sections, we will summarize these sex differences in clinical asthma in humans and elaborate on the effects of cycling estrogen levels on asthma severity and incidence in women. Although other sex hormones such as androgens and progesterone influence the immune system and the action of estrogen in cells, we will focus this review on the studies of estrogen on the immune system. Our emphasis here will be the effect of estrogen on innate immune cells, with some discussion of the adaptive immune system. We will describe the estrogen receptors (ERs) and their regulation of gene expression. We will then review recent insights from animal models regarding the impact of estrogen signaling on allergic inflammation, the various cell types involved, and how this may account for the sex difference observed in asthma.

## Incidence of Asthma and Allergy is Greater among Women than Men: Sex Differences in Asthma

The onset and severity of asthma and allergies typically exhibit sex differences, although the results of clinical surveys differ somewhat depending on geography, likely highlighting environmental, cultural, and dietary factors. Virtually all atopic allergies affect boys more than girls in childhood. Numerous studies that have focused on questionnaire-based and clinician-diagnosed school children with atopic dermatitis have reported a male-dominant incidence that persists from birth until 6–11 years of age (15–17). Conversely, a Danish study reported that while the onset of atopic dermatitis was slightly delayed in girls (2.5 years of age) compared to that in boys (2 years of age), the overall prevalence did not differ by sex (18). The perceived prevalence of peanut allergy in Great Britain exhibits female predominance in general, although after age 15, that trend is reversed (19). A Norwegian study found girls exhibit a higher incidence of food allergies than boys after 18 months of age (20). Allergic rhinoconjunctivitis seems to preferentially affect males beyond the age of 13 (21). The onset of puberty, however, reverses that trend for most allergic disorders, including asthma (22). Women over the age of 25 account for >62% of hospitalizations due to acute asthma attacks and, in 2009, accounted for 64% of asthma-related deaths (23, 24). Roughly 40% of women experience premenstrual exacerbations of asthma and women are more likely to be non-responsive to corticosteroid therapy (25, 26).

Asthma symptoms in women seem to change with various life-stage factors such as menstruation, pregnancy, and menopause (27–30). The incidence of allergy and asthma peaks in women after puberty and strongly subsides with age (31). Roughly 33–52% of asthmatic women report premenstrual worsening of symptoms (32–35). Almost 50% of women hospitalized for asthma symptoms are identified as premenstrual (36). Furthermore, spikes in menstrual hormones correlate with impaired peak expiratory flow rate and thus reduced lung function (37). Lung function is generally worse during the late follicular phase, when estrogen levels are highest (38). Abrupt estrogen spikes are alleviated with oral contraceptive use, which was shown to stabilize asthma symptoms in women (39). Yet, the worsening of symptoms with oral contraceptive use has also been reported (40). Pregnancy causes a large increase in circulating estrogen that starts with ovum fertilization and falls to prepregnancy levels shortly after delivery. Approximately 70% of asthmatic women report alleviation of symptoms during pregnancy (28, 35, 41–43). Symptom severity seems to return to normal within 3 months postpartum. Interestingly, postpartum symptom severity worsens with increasing number of births (44). Estrogen levels drop during menopause and are lower in asthmatic women than in healthy women (45). Menopause typically results in exacerbated asthma symptoms and leads to the development of new asthma in previously unaffected women (46). Hormone replacement therapy (HRT) increases estrogen levels and alleviates symptoms in postmenopausal asthmatic women (45). However, HRT use has also been linked to a 19% increased risk of hospitalization per year of use in menopausal women with asthma (47). Furthermore, rates of new asthma diagnosis have been reported to increase after HRT initiation but diminish with cessation of HRT (48). Because asthma comprises several distinct endotypes, this likely contributes to the seemingly conflicting outcomes of oral contraceptive and HRT use in women.

These survey studies collectively suggest that estrogen levels correlate with symptom severity and likely contribute to sex differences in asthma. In general, women have a greater incidence and severity of asthma than do men (49–51). However, the alleviation of symptoms in women during stages of life that are accompanied by high levels of circulating estrogen suggests that the inflammatory function of estrogen may be dose-specific. This idea is echoed in *in vitro* and animal studies covered later in this review. To understand how estrogens impact the immune system, we will first give some background on ER biology in the next section.

## Estrogen Receptor Biology and Isoforms

Estrogen signaling regulates reproductive physiology and gene expression in many tissues and cell types. Not surprisingly, the failure to regulate estrogen signaling is associated with a variety of human diseases, including breast and endometrial cancer, cardiovascular disease, osteoporosis, and Alzheimer’s disease [reviewed in Ref. (52)]. Like all hormones, estrogen readily penetrates the cell membrane. In the cytosol, it encounters ERs, triggers their dimerization, and liberates them from an inactive complex with heat shock protein (HSP)90 (53). The ERs then translocate to the nucleus and engage estrogen response elements (EREs) on target-gene promoters (53). However, this signaling network exhibits several layers of regulatory complexity that result in pleiotropic effects on various tissues and cell types. This diversity in the biological functions of estrogen is achieved through the expression of several ER isoforms that have the capacity to interact with various transcriptional coactivators and corepressors as well as transcription factors to elicit an array of cellular responses. In fact, estrogen is known to elicit non-genomic effects on cells via membrane-bound receptors that crosstalk with an array of cellular signaling networks (54, 55). Furthermore, phosphorylation of the nuclear ERs can mimic ligand binding and thus induce ligand-independent responses (56–58). In the following section, we will discuss the current understanding of the estrogen signaling pathway.

**Figure 1:**
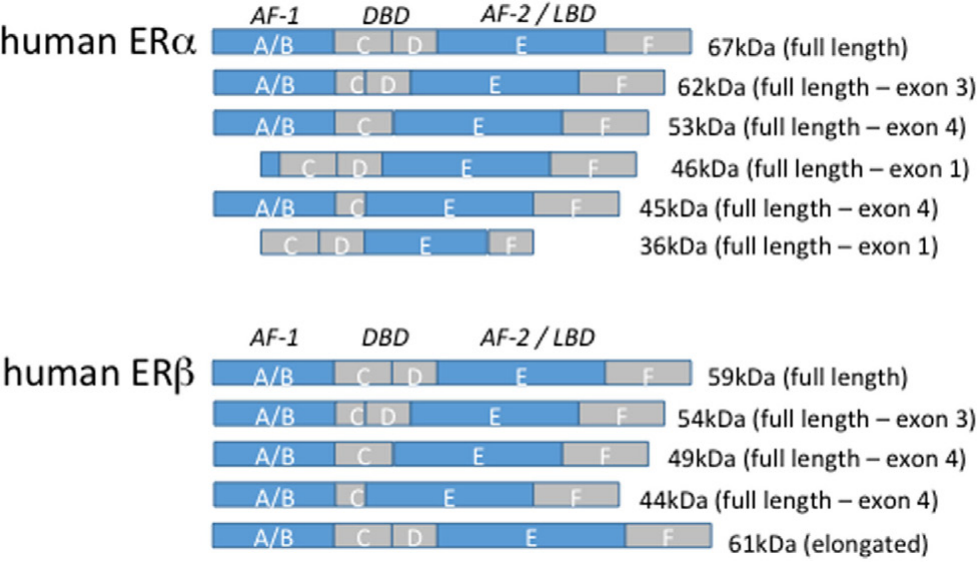
**Estrogen receptor isoforms**. The domain organization of human estrogen receptor α and β isoforms are illustrated above. The size of the full-length, truncated, and elongated isoforms is indicated.

The nuclear ERs exist in two main isoforms termed ERα and ERβ, which are part of a large superfamily of type I nuclear receptors. The nuclear receptor superfamily members exhibit a conserved structure consisting of regions A–F (Figure 1). ERα and ERβ both contain an N-terminal activation function-1 (AF-1) domain within regions A and B, a zinc-finger containing a DNA-binding domain in the centrally located region C, and a C-terminal AF-2 domain within regions E/F that facilitates dimerization, association with Hsp90, and ligand binding via the ligand-binding domain (59). Region D consists of a hinge between the N- and C-terminal halves of the receptor. In the absence of ligand, ERα and ERβ are bound to heat shock proteins that restrict their activity. Ligand binding, however, releases the receptors from this complex and allows their engagement of target gene promoters. Several serine and tyrosine residues on the receptors are also subject to phosphorylation that enhances receptor activity [reviewed in Ref. (60)]. Upon ligation, the DNA-binding activity of the AF-1 domain and the dimerization activity of the AF-2 domain facilitate transcriptional regulation through recruitment of >40 different coactivators, including histone acetyltransferases, ubiquitin ligases, arginine methyltransferases, and transcription factors.

Estrogen receptor α consists of the full-length 66 kilodalton (kDa) isoform and alternatively spliced truncated 36 and 46 kDa isoforms that result from internal ATG transcription start codons. The 46 kDa isoform lacks the AF-1 domain, and 36 kDa isoform lacks both the AF-1 and AF-2 domains preventing efficient transcriptional activity but permitting heterodimerization with the full-length receptor (61, 62). Human macrophages primarily express the 46 kDa isoform of ERα and to a lesser extent the full-length 66 receptor and the 46 kDa isoform is estrogen inducible (63). Furthermore, the transition of monocytes along the monocyte-macrophage axis is accompanied by an upregulation of the 46 kDa ERα (63). Both isoforms can localize to the membrane. The 5′ flanking region of the 36 kDa isoform contains several putative transcription factor-binding sites, including NF-κB, glucocorticoid receptor (GR), specificity protein (SP)1, and activator protein 1, but is suppressed by full-length 66 kDa ERα (64). ERα-36 is the only ERα isoform expressed in human peripheral blood monocytes and suppresses proinflammatory cytokine production in response to LPS (65). Other truncated isoforms have been identified in a variety of human cancers and rat cells although their expression in healthy human tissue is poorly understood (66, 67). ERβ consists of at least five different truncated isoforms that lack either the AF-1 or AF-2 domain.

## Transcriptional Regulation by ERs

The conventional linear pathway of nuclear receptor signaling whereby ligated hormone receptors regulate gene expression at well-defined promoter elements has been revised in recent years. When bound to ligand, ERα and ERβ form either homo- or heterodimers and engage EREs on target gene promoters [reviewed in Ref. (68)]. Intriguingly, although EREs can be functional even as far as 2 kilobases (kb) from their target promoter and exhibit activity either upstream or downstream from the target gene, approximately one-third of estrogen-regulated genes in humans lack EREs altogether (69). Rather, association of ERs with SP-1 mediates indirect ER–DNA binding and utilizes the coactivator-recruiting activity of ERs to drive gene expression independently of EREs. This form of ERE-independent gene induction has been identified for low-density lipoprotein receptor, endothelial nitric oxide synthase (eNOS), c-*fos*, cyclin D1, and the retinoic acid receptor 1α (70–74). Other transcription factors, such as NF-κB, *c-jun*, and cyclic adenosine monophosphate (cAMP) response element-binding protein have also been shown to recruit ERs for ERE-independent sites (75). Interestingly, human ERα and ERβ seem to have opposing effects on these promoters, with ERα-enhancing and ERβ-repressing gene expression. This phenomenon may reflect a defective AF-1 domain in human ERβ (76).

The canonical steroid receptor coactivator (SRC) family associated with ERs is termed the SRC/p160 family. The members of this family are referred to by various names in the literature but consist of SRC-1 (also termed NCoA-1), SRC-2 (also termed TIF2, GRIP1, or NCoA-2), and SRC-3 (also termed RAC3, pCIP, ACTR, AIB1, TRAM-1, or NCoA-3). These coactivators contain a nuclear receptor box that consists of an LXXLL motif that fits within the hydrophobic groove formed by the 12 α-helices of the AF-2 domain (77). Unligated or tamoxifen-bound ERs can also interact with nuclear receptor corepressors N-CoR and SMRT, which inhibit the recruitment of SRC/p160 and thus repress transcription at ERE sites (78).

## Post-Translational Modifications of ER

Estrogen receptor α and ERβ contain conserved serine residues that are phosphorylated in response to ligand binding as well as independently of ligand binding. Serines-104, -106, and -118 have been shown to be phosphorylated by cyclin-dependent kinases after estrogen binding (79). These sites reside within the AF-1 domain and enhance ER activity. In addition, ligand-independent activation of the ERs by growth factors and cyclic AMP has been described. In fact, Erk1/2 can directly phosphorylate serines-104 and -118 within the AF-1 domain of ERα and induce dimerization and activation in response to either epidermal growth factor or estrogen signaling (60, 80, 81). Ribosomal S6 kinase and protein kinase B (Akt) have been shown to phosphorylate serine-167 in response to growth factor signaling (82, 83). Protein kinase A activation and cAMP treatment of cells lead to phosphorylation of serine-236, which resides within the DNA-binding domain (84, 85). A tyrosine residue (Tyr-537) within the AF-2 domain is also phosphorylated in ERs and serves as a docking site for the Src homology-2 domain of c-Src (86). The general outcome of these phosphorylation events is enhanced receptor activity. Several groups have compared mutants with alanine substitutions at these serine sites to wild-type receptors using ERE reporter assays and found that the inability to phosphorylate these sites translates to impaired target gene expression (56, 80, 87, 88).

Estrogen receptors not only regulate reproductive and developmental physiology but also participate in hormone-independent signaling by interacting with growth factor and other cell-signaling networks. As described earlier, ER signaling is regulated by several factors, including the relative abundance of receptor isoforms, interactions with coactivators/corepressors, and DNA-remodeling proteins, receptor phosphorylation, and inputs from other signaling networks. This variety in regulation permits profound functional versatility and provides an avenue for hormonal influence over many biological pathways and processes, like allergic inflammation. More research is required to ultimately understand how ER signaling interacts with non-hormonal pathways. With respect to asthma, there is an imperative for greater understanding of the pathophysiology of various endotypes that comprise the disease so that the potential contribution of ER signaling and gene induction can be clarified.

## Effect of Estrogen on the Innate Immune System

In the last few decades, our understanding of the profound effect of sex hormones on leukocyte biology has expanded dramatically [reviewed in Ref. (89)]. Castration and hormone replacement studies in mice have been instrumental in connecting anecdotal evidence of sex differences in humans to the underlying cellular mechanisms. Cell-specific knockout mice have provided the essential tools to isolate the role of individual ER isoforms and dissect their involvement in the inflammatory process. Studies in selective ER knockout animals have revealed a complex interplay between receptor isoforms, sex hormone levels, cell types, and biological outcomes. The following section will summarize the current understanding of estrogen signaling in cells of the innate immune system that are relevant to the pathogenesis of allergic inflammation in the lung during asthma.

## Macrophages

In recent years, the role of macrophages in allergic inflammation has garnered increased attention. In asthma, alveolar macrophages have emerged as important cellular mediators of inflammation and tissue remodeling [reviewed in Ref. (90–92)]. Allergic asthma is considered to be a Th2 disease and is thus accompanied by IL-4/13-polarized M2 alveolar macrophages in the airways. Intratracheal instillation of alternatively activated M2 macrophages prior to ovalbumin (OVA) challenge results in enhanced eosinophil and effector T cell infiltration into the BAL in mice (93). Macrophage depletion studies have revealed a dual role for alveolar macrophages in eliciting and containing allergic lung inflammation in mice (94). Resident alveolar macrophages are inherently immunosuppressive and secrete anti-inflammatory factors, such as transforming growth factor β (TGFβ) and IL-10 (95, 96). In the allergic setting, however, the bronchoalveolar space is infiltrated by inflammatory M2-polarized macrophages that facilitate inflammation by secreting chemokines, such as chemokine c–c motif ligand 2 (CCL2), CCL17, Ym1, and found in inflammatory zone 1 (Fizz1 or Relmα) (97). Depletion of these macrophages in mice results in impaired IL-5 production, eosinophil influx, and airway remodeling (97). In the house, dust mite model of mouse allergic lung inflammation, female mice have more M2-like (Ym1^+^) cells, whereas males have more M1-like (interferon regulatory factor 5) cells in histological lung cross-sections (98). Likewise, female mice exhibit more eosinophils, effector T cells, and M2-polarized macrophages in the lungs than do males in the OVA model of lung inflammation (93). Furthermore, the production of lipid inflammatory mediators like leukotrienes via 5-lipoxygenase is enhanced in female leukocytes compared to that in male leukocytes, but this difference is due to suppression of the pathway by testosterone (99). This central role of macrophages in allergic lung inflammation has warranted studies addressing the potential influence of sex hormones on macrophage polarization and inflammatory cytokine production.

**Figure 2:**
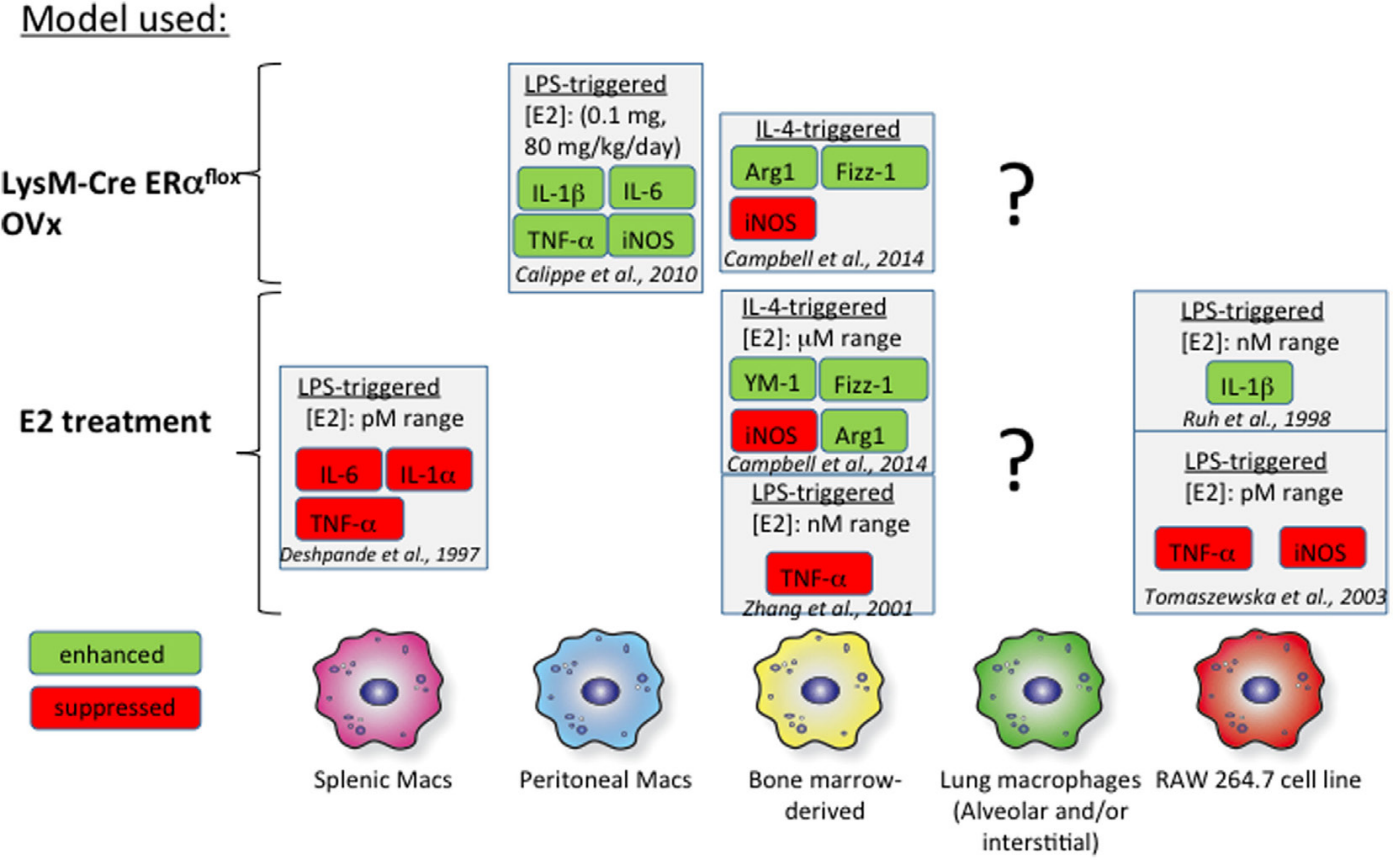
**Effects of estrogen *in vitro* on macrophages**. The effects of estrogen on macrophages from various tissues are illustrated above. The findings of several studies are summarized and referenced in the figure. The effects of estrogen on proinflammatory mediators are highly dependent on the tissue of origin. There is a general lack of data focusing on the impact of estrogen signaling on alveolar macrophages, indicated by the question marks.

Bone marrow-derived macrophages (BMMs) have served as a proxy *in vitro* model for studies addressing the effect of estrogen on macrophage biology. Several groups have measured macrophage cytokine production in response to interferon γ (IFNγ) or IL-4 in the context of either exogenous estrogen treatment or macrophages from ER knockout mice (Figure 2). IL-4-induced M2 polarization is enhanced with the addition of exogenous estrogen at micromolar doses in mouse BMMs (100). Estrogen not only increases arginase 1 (Arg1) activity and Fizz1 expression in IL-4-polarized BMMs but also suppresses inducible NOS (iNOS) expression in IFNγ/lipopolysaccharide (LPS)-treated BMMs. Furthermore, these effects were determined to be mediated through ERα signaling, as they were mimicked with propyl pyrazole triol (PPT), a selective ERα agonist, and not diarylpropionitrile (DPN), a selective ERβ agonist. Likewise, BMMs from LysM-CRE ERα*^*flox/flox*^* mice fail to upregulate Arg1 and Fizz1 in response to IL-4 and exhibit enhanced iNOS responses to IFNγ/LPS. Physiological doses of estrogen (picomolar to nanomolar range) suppress LPS-induced tumor necrosis factor α (TNFα) expression in mouse BMMs (101). BMMs from ovariectomized (OVx) mice exhibit decreased TGFβ1 mRNA, but exogenous treatment with estrogen rescues this phenotype, suggesting that estrogen may contribute to tissue remodeling in allergic inflammation through TGFβ1 induction (102).

Studies addressing estrogen signaling in splenic, wound-derived, and peritoneal macrophages have revealed heterogeneity in responses between macrophages obtained from different sources (Figure 2). Signaling through ERα with 1 μM PPT in murine wound-derived macrophages enhances M1 (iNOS and TNFα) and M2 (Arg1 and Ym1) responses following polarization with IFNγ/LPS and IL-4, respectively (100). Conversely, signaling through ERβ with 1 μM DPN has the opposite effect on M1 and M2 wound-derived macrophages. Thioglycollate elicitation of peritoneal macrophages is impaired in OVx mice replenished with estrogen (103). However, these thioglycollate-elicited macrophages in estrogen-replenished OVx mice are proinflammatory in nature and express more IL-1β, IL-6, iNOS, and TNFα than do cells from OVx mice. These effects of estrogen were determined to arise from ERα-mediated inhibition of phosphoinositide 3-kinase. Splenic macrophages treated with lower doses of estrogen (picomolar range) exhibit impaired LPS-induced TNFα production compared to that in untreated cells. These studies highlight a lack of uniformity in study design but also suggest that macrophages exhibit heterogeneity with respect to estrogen signaling. Furthermore, macrophage subsets found from different tissue sources exhibit unique phenotypes, complicating our ability to relate studies that use mouse BMMs or peritoneal and splenic macrophages to alveolar macrophage biology (104, 105). Thus, more *in vivo* studies are needed that examine the effect of estrogen on alveolar macrophages in the setting of allergic inflammation. A stable mouse or human alveolar macrophage cell line from would also provide a more appropriate *in vitro* model for understanding lung inflammation.

## Dendritic Cells

Dendritic cells (DCs) are essential initiators of the adaptive immune responses that facilitate sensitization to allergens. Estrogen is intricately involved in DC function from differentiation to maturation and proinflammatory cytokine production. GM-CSF-induced DC differentiation from mouse bone marrow precursors is dramatically impaired when conducted using hormone-free serum in the culture medium (106). Adding estrogen (picomolar range) into the culture medium of these cells restores DC differentiation and responsiveness to LPS, as well as their capacity for T cell priming. Bone marrow from ERα^−/−^ mice is defective in generating DCs with GM-CSF, although the addition of 10 nm estrogen partially resolved that, suggesting that other ERs are also important in DC differentiation (106). In fact, GM-CSF-generated bone marrow-derived CD11b^+^CD11c^+^ DCs express both ERα and ERβ (107). A CD11c^+^CD11b^int^ subset that emerges from 100 pM estrogen-enhanced GM-CSF-generated bone marrow-derived DCs expresses langerin and exhibits other features of Langerhans’ cells (107). Thus, estrogen has an enhancing effect on DC differentiation from myeloid progenitors.

The effects of estrogen on DC maturation and cytokine production have been addressed in several *in vitro* and *in vivo* models. Upon encountering pathogen-associated molecular patterns (PAMPs), DCs mature by downregulating endocytosis and upregulating the antigen presentation apparatus along with cytokine production. Human plasmacytoid DCs (pDCs) exhibit profound sex differences when stimulated with the toll-like receptors 7/8 ligand R-848 with pDCs obtained from premenopausal women being more responsive by expressing IFNα and TNFα compared to age-matched males (108). Estrogen therapy in menopausal women dramatically increased the cytokine responsiveness of blood pDCs to TLR ligation, and this was recapitulated in an OVx mouse model (108).

Plasmacytoid DCs are known to be potent T_h_2-priming antigen-presenting cells, and they are more abundant in the blood of patients with asthma compared to healthy individuals (109, 110). IL-4-mediated depletion of pDCs is impaired in patients with asthma compared to healthy controls, despite no measurable difference in IL-4Rα expression (109). Conversely, pDCs also drive tolerance to allergens in OVA-challenged mice, and their depletion exacerbates eosinophil influx and tissue remodeling in the lung (111). Further, the adoptive transfer of pDCs back into depleted mice rescues this exacerbation. The adoptive transfer of pDCs cultured with house-dust mite allergen into mice results in an expansion of FoxP3^+^ Tregs as well as IL-10 production following allergen challenge (112). Human monocyte-derived DCs treated with ~70 μM estrogen exhibit induction of MCP-1, IL-8, and IL-6 but an unaltered immature phenotype with respect to major histocompatibility complex II (MHCII) and costimulatory molecule expression (113). Splenic DCs, however, respond to estrogen (10 nM – 1 μM) by upregulating CD40 and MHCII (114). At high doses (in the micromolar range), estrogen downregulates endocytosis in splenic DCs and promotes survival as well as IL-6 and IL-10 productions. Likewise, estrogen enhances T-cell proliferation in culture with LPS-activated DCs, suggesting that it promotes the capacity of mature DCs to stimulate T-cell responses. Further, estrogen treatment (1 nM) of mouse bone marrow enhances GM-CSF-induced DC differentiation and LPS/CpG-induced IL-12 production (115). *In vivo* experiments that focus on the effects of estrogen on DC-mediated T-cell priming are lacking. However, one group found that administration of estrogen to OVx mice enhanced Th1 responses to a subcutaneous OVA challenge and that the response required ERα expression in the hematopoietic compartment (116). The adoptive transfer of estrogen-treated DCs (~73 nM) was shown to alleviate inflammation in experimental allergic encephalomyelitis (EAE) (117). In that model, estrogen administration in Lewis rats impaired the capacity of DCs to prime Th1 and Th2 T-cell response.

Like macrophages, these studies suggest that estrogen has differing effects on DCs, depending on their tissue of origin as well as the species being studied. Bone marrow- and monocyte-derived DCs exhibit enhanced inflammatory cytokine production and toll-like receptor ligand responsiveness with estrogen treatment. In splenic DCs, estrogen promotes the capacity for T-cell priming. In the EAE model, however, DC function seems to be less inflammatory with estrogen treatment (118). More studies are needed to discern the various dose-dependent effects of estrogen on DC subsets from different tissues. Studies in asthma are particularly important because lung DCs have a major role in priming T cells against allergens (119, 120).

## Eosinophils

Eosinophils are a hallmark cell type of allergic inflammation. These cells are recruited from the blood by IL-5, which is produced in response to epithelial TSLP, IL-25, and IL-33. Eosinophils are recruited as mature cells and elicit antihelminth immunity and inadvertent tissue damage by degranulation. Eosinophil granules contain several different effector molecules. Major basic protein, eosinophil peroxidase, eosinophil cationic protein, and eosinophil-derived neurotoxin are among the granular content of eosinophils. They have both distinct and overlapping effects on target cells. In general, these granular proteins exert toxic effects on target cells by a variety of mechanisms, including pore formation, ribonuclease activity, and the production of reactive oxygen species. Eosinophils are also significant sources of cytokines, such as IL-4 and IL-13, and thus promote allergic inflammation once these cytokines are released.

Estrogen has been shown to regulate eosinophil migration, adhesion, survival, and degranulation. Its influence on eosinophil biology became clear as early as the 1960s, when it was first reported to induce eosinophilic infiltration into the rat uterus as a hallmark of uterine development and tissue remodeling (121–123). Additionally, the number of eosinophils in the uterus was found to follow the estrous cycle, peaking at the estrus phase and dipping at the diestrus phase (124). Uterine eosinophils are virtually absent from OVx animals and late in pregnancy.

**Figure 3:**
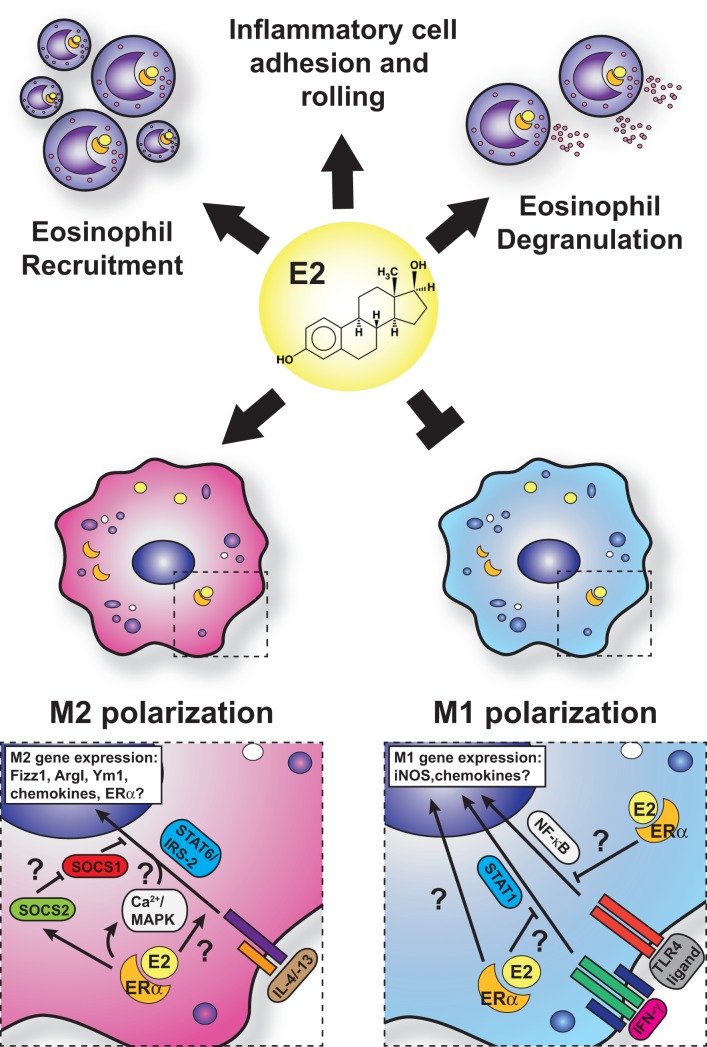
**Current model for estrogen signaling in polarization in macrophages in the setting of allergic lung inflammation**. The general consensus is that estrogen enhances M2 polarization while suppressing M1-polarization in macrophage. However, mechanistic insights are lacking, and questions remain regarding the interface of ERα and IL-4 signaling.

Eosinophils are a key cell type involved in the pathogenesis of asthma and allergy and are known to express ERα and membrane-bound G-protein-coupled receptor (GPR-1) in mice and humans (125, 126). The sex differences associated with allergic inflammation, and in particular asthma, are most apparent with the varying degrees of eosinophilia in asthma models in male and female mice. Female mice exhibit greater eosinophilic infiltration into the bronchoalveolar and parenchymal spaces than do male mice, coincident with increased allergen-specific IgE, increased cluster of differentiation 4 (CD4)^+^ T- and B-cell accumulation, and impaired lung function (127, 128). Treatment of allergen-challenged mice with ERα antagonists, such as tamoxifen and ICI 182,780, dramatically reduces BAL eosinophil numbers and Th2 cytokines, such as IL-5 and IL-13 (125, 129). Furthermore, exogenous estrogen dose dependently induces both IL-5 and IL-13 in mediastinal lymph node cultures from allergic animals. Intriguingly, ovariectomy prior to OVA sensitization impairs eosinophil influx, which is restored with exogenous estrogen (129). However, ovariectomy of mice after sensitization to OVA exhibit enhanced eosinophilia, suggesting that estrogen may on the one hand promote T-cell priming against allergens but on the other control eosinophil infiltration after challenge. Conversely, Dimitropoulou et al. (130) reported a dose-dependent reduction in eosinophil infiltration into the BAL of estrogen-treated, OVA-challenged, OVx mice. These discrepancies may reflect differences in experimental approaches and doses of estrogen replacement used. Estrogen administration triggers eosinophil degranulation in rats *in vitro* and *in vivo* (131). Although in a model of acute thioglycollate-induced sterile peritonitis, estrogen plays an inhibitory role in eosinophil influx and survival in mice (132). The administration of estrogen in OVx mice reduces peritoneal eosinophils and IL-5 production in this model, and these are restored in ERα gene knockout mice. Furthermore, administration of the GPR-1 agonist, G-1, to C57BL/6 and BALB/c mice subjected to an OVA-induced allergic inflammation model reduces lung eosinophilia, IL-5, and eotaxin production through induction of IL-10 (133). However, human eosinophils exhibit enhanced eotaxin-induced chemotaxis and survival with G-1 treatment (113). Thus, estrogen impacts eosinophils in different ways depending on the receptors engaged, the species used, the tissue in which the signaling is taking place, and whether the cells are analyzed *in vivo* or *in vitro*. Some of the known effects of estrogen on eosinophils that would increase allergic inflammation are summarized in Figure 3.

## Mast Cells

Mast cells are detectable in the sputum of asthmatic patients, but sex differences with respect to mast cell function have not been adequately explored (134). Nevertheless, their numbers increase and decrease in female reproductive tissues with the estrous cycle, suggesting that estrogen regulates mast cell migration and biology. Mast cells from rat and human cell lines as well as mouse bone marrow-derived mast cells express ERα but not ERβ (135). In rat mammary glands, mast cells accumulate during the estrus and diestrus phases of the estrous cycle (136). After ovariectomy, mast cells decrease dramatically, but their numbers are restored with estrogen replacement. Estrogen induces IgE-dependent and -independent mast cell degranulation in rats (135, 137). These effects are dependent on Ca^2+^ and thus are non-genomic although the expression of GPR-1 was not evaluated in this study. Mouse models of lung allergen challenge clearly suggest that mast cells orchestrate inflammation, but they do not accumulate in BAL (138). To our knowledge, no studies have investigated the effect of sex on the contribution of mast cells to allergic inflammation in mice.

## Airway Epithelial Cells

The airway epithelium provides front-line defense against invading pathogens and environmental insults in the lung by balancing gas exchange with innate immune function. Airway epithelial cells express pattern recognition receptors that enable them to detect and respond to PAMP and danger-associated molecular pattern by secreting cytokines and chemokines to drive inflammation. In asthma, the airway epithelium recruits innate and adaptive immune cells via cytokines, such as IL-25 and IL-33, and chemokines, such as CCL2, CCL17, and CCL20. Furthermore, these cells secrete TGFβ, which promotes tissue remodeling. Human bronchial epithelial cells express both ERα and ERβ and are responsive to estrogen exposure. Treatment of these cells with 10 nM estrogen induces endothelial NOS expression and nitric oxide production that results in bronchodilation (139). Similarly, the same dose of estrogen relaxes human airway smooth muscle and thereby induces bronchodilation (140). Estrogen replacement in OVx mice significantly reduces acetylcholine responsiveness (141). These studies suggest that estrogen is protective against airway constriction and thus may be therapeutic in asthma. However, estrogen treatment of human airway epithelium also induces MUC5B secretion, which may contribute to airway lumen obstruction (142).

Currently, very little is known regarding the effect of estrogen on proinflammatory cytokine production by airway epithelial cells. In patients with cystic fibrosis, estrogen treatment reduces epithelial IL-8 production, which is thought to predispose women to *Pseudomonas* infection (143). Thus, estrogen may be a double-edged sword with respect to its bronchodilation and inflammatory properties. The outcome on lung function is likely the net result of a balance between increased allergic inflammation and smooth muscle relaxation/increased bronchodilation.

## Effect of Estrogen on the Adaptive Immune System

Estrogen signaling has profound effects on adaptive immunity. Consequently, women are generally at greater risk than men for pathology associated with immune responses to infection, autoimmune diseases, and allergies. The underlying cellular basis for these sex differences is currently being evaluated. Estrogen signaling is known to be important for thymic development and antibody production, although pregnancy levels of estrogen induce thymic attrition and suppress proinflammatory responses in mice and humans (144, 145). Thus, physiological estrogen levels can be functionally grouped into several categories that reflect various developmental stages of life. Concentrations of circulating estrogen vary between 40–152 pM and 1–150 nM in menopausal and pregnant women, respectively [reviewed in Ref. (14)]. Research approaches, including ER knockout, overiectomy of animals followed by estrogen replacement, and comparisons of estrogen responses in healthy and diseased lymphocytes, have provided important insights into the cellular basis for sex differences. This section will briefly summarize our current understanding of how estrogen signaling affects T and B lymphocytes and the consequences that may contribute to sex differences in health and disease.

## T Cells

The effects of estrogen on T cells are complex and context and dose specific. As discussed later, estrogen has the capacity to enhance Th1, Th2, and Treg responses. At concentrations found during pregnancy (1–150 nM), estrogen promotes IFNγ production by CD4^+^ T cells. At those same concentrations, estrogen enhances *ex vivo* production of IL-10, IFNγ, and TNFα by proteolipid-specific CD4^+^ T cells from patients with multiple sclerosis compared to that by cells from healthy patients and cells exposed to proteolipid alone (146). Interestingly, higher doses of estrogen seem to inhibit TNFα production but have no effect on IL-4 production. Similarly, replacement of estrogen at pregnancy-level concentrations in OVx mice suppresses T cell-mediated type IV hypersensitivity reaction (147, 148).

Non-obese diabetic (NOD) mice exhibit sex differences in the cytokine profile of islet-infiltrating CD4^+^ T cells, as females produce more IFNγ and less IL-4 than do males (149). Estrogen replacement in OVx NOD mice, at levels present during pregnancy, enhances this IFNγ production in islet infiltrates. Lambert et al. (150) reported similar enhancement of IFNγ production by ConA-activated splenic CD4^+^ T cells from C57BL/6 mice stimulated with pregnancy-level concentrations of estrogen. Likewise, subcutaneous OVA sensitization in OVx C57BL/6 mice leads to an estrogen-dependent increase in IFNγ^+^ CD4^+^ T cells within the draining lymph nodes, an effect that is abrogated in ERα knockout mice (116). Conversely, estrogen at pregnancy concentrations increased the expression of IL-4 and GATA-3 in a dose-dependent manner by CD3/CD28-cultured splenic T cells from C57BL/6 mice but not in those from ERα knockout mice (151). Concentrations of estrogen measured during pregnancy also promote FoxP3 and CD25 expression on mouse T cells *in vivo* and *in vitro* in an ERα-dependent manner (152). Furthermore, ERα expression in T cells is required for estrogen-mediated protection in the mouse model of EAE via the reduction of IFNγ and IL-17 expression by CD4^+^ T cells (153). Indeed, patients with multiple sclerosis show improvement during pregnancy and this is likely due to elevated estrogen (154). Collectively, these findings suggest that concentrations of estrogen found during pregnancy enhance IFNγ, IL-10, and IL-4 production by CD4^+^ T cells and thus may protect the fetus against Th17-mediated immunopathology.

## B Cells

In general, estrogen enhances B-cell maturation and antibody production. Cervical mucus IgA levels peak in cycling women prior to ovulation and are increased by oral contraceptives (155). Human blood mononuclear cells cultured with sheep red blood cells (SRBC) likewise produce more anti-SRBC antibodies in response to increasing doses of estrogen, an effect that is ablated with tamoxifen (156). Interestingly, in this model, estrogen was found to interfere with CD8^+^ T-cell suppression of B-cell maturation. Indeed, estrogen enhances IgG and IgM production in humans, and neutralization of IL-10 impairs this response (157, 158). B cells from patients with systemic lupus erythematosus produce increased anti-dsDNA antibodies in response to estrogen also because of IL-10 production (159). Additionally, high concentrations of estrogen (micromolar range) increase the amount of antibody produced by pokeweed mitogen-stimulated mouse peripheral blood mononuclear cells (145). Importantly, estrogen and estrogenic compounds enhance IgE production by mouse splenocytes and thereby may contribute to allergic inflammation (160).

## Future Directions

To date, studies addressing the role of estrogen and other sex hormones in allergy and immunity have been confronted with major experimental limitations, resulting in a lack of consensus over confounding data. *In vitro* experiments utilizing exogenous treatment of cells with agonists or antagonists to ERs are a good starting point but hardly address how these cells behave in the context of the inflamed lung *in vivo*. Furthermore, there must be a uniformity regarding the dose, duration, and delivery of estrogen treatment in either *in vitro* models or estrogen replacement *in vivo* models. Exceeding normal physiological doses (into the micromolar range) significantly contributes to confounding reports as estrogen exhibits dose-specific properties and has the potential to affect an ever-growing spectrum of cellular pathways. There is also an inherent difficulty in gaining mechanistic insights from either estrogen replacement in OVx mice or from global ER knockout mice since there are systemic consequences to these animal models and allergic responses are complex multicellular processes. Thus, the development of more sophisticated mouse models is imperative to address the impact of estrogen on the various phases of allergic inflammation and on the key specific cell types in a controlled manner. For this purpose, the employment of inducible Cre recombinase under cell-specific promoters to target floxed ER alleles for knockout would be very helpful.

Many questions remain to be addressed regarding the role of estrogen in allergic inflammation. As indicated in Figure 2, the impact of estrogen on alveolar macrophages has been overlooked. Since these cells are important modulators of allergic lung inflammation, more attention should be focused on how estrogen affects their biology in the setting of allergic lung inflammation. The estrogen-mediated effects of changes in macrophage function, tissue remodeling and fibrosis, as well as eosinophil and monocyte recruitment are yet to be explored. Furthermore, the potential recruitment of inflammatory monocytes and the impact of estrogen on their trafficking and polarization have yet to be addressed. The respective contribution of ERα, ERβ, and GPR-1 to the function of innate and adaptive immunities in allergic inflammation needs to be addressed using a targeted and inducible knockout *in vivo*. Furthermore, the potential role of ERs in steroid resistance has major therapeutic promise. Whether ERs interfere with GR signaling in hard-to-treat asthma that mostly affects women is unknown. Alveolar macrophages may play a role in this pathology, since alveolar macrophages from patients with asthma exhibit relative resistance to dexamethasone compared to healthy individuals (161–164).

## Concluding Remarks

Allergic disorders exhibit considerable sex-specific differences in prevalence, severity, age of onset, and clinical outcomes. These differences likely reflect the effects of sex hormones on the immune system, as well as other lifestyle factors. Adult asthma is clinically dominated by women, and the contribution of estrogen to this sex difference is becoming clear. However, the apparent lack of uniformity and consistency between experimental approaches used to address this issue has led to numerous confounding and conflicting results. Furthermore, asthma is a heterogeneous disease, and a one-size-fits-all approach to therapy is no longer considered reasonable. Therefore, it is imperative that the several different endotypes that comprise asthma be fully characterized and suited with appropriate rodent and *in vitro* models that will enable us to address potential therapeutic avenues. The Th2 paradigm used in animal models needs to be reconsidered, as it addresses only 50% of clinical asthma. To date, the most common treatments for asthma are inhaled glucocorticoids for suppression of inflammatory gene expression and β2-adrenergic receptor agonists for inhibition of bronchoconstriction. Women are exceptionally burdened with hard-to-treat, corticosteroid-resistant, neutrophilic asthma (165–167). The potential interactions between estrogen and glucocorticoid receptors have not been addressed in corticosteroid-resistant asthma despite their sharing coactivator binding partners. Furthermore, the persistent use of glucocorticoids results in significant side effects, such as cataracts and osteoporosis among others (168, 169). Neutralizing antibodies to IL-4Rα and IL-13, such as dupilumab and lebrikizumab, respectively, do show promise in clinical effectiveness but only in select patients with typical Th2-type asthma (170, 171). There is now an apparent shift in thinking that focuses on matching particular treatments to patients presenting with the appropriate biomarkers. This approach, however, requires extensive research with appropriate models. Estrogen signaling affects virtually every cell of the immune system. It is thus an appealing avenue for therapy but must certainly be carried out in a cell-specific and targeted manner that does not interfere with reproductive physiology. To achieve this goal, we must revise animal models to address the role of estrogen in allergic inflammation in a more reductionist manner. Cell-specific ER knockout models are essential for understanding the effect of estrogen signaling on a given cell type without dramatically altering the biology of the animal. The development of promoter-specific inducible CRE models permits such approaches. Such models, coupled with *in vitro* biochemical studies, may help to identify specific interactions between an ER isoform and a coactivator that facilitate enhanced allergic inflammation in women and can thus be targeted therapeutically.

## Conflict of Interest Statement

The authors declare that the research was conducted in the absence of any commercial or financial relationships that could be construed as a potential conflict of interest.
